# A Genome-Wide Association Study for Diabetic Retinopathy in a Japanese Population: Potential Association with a Long Intergenic Non-Coding RNA

**DOI:** 10.1371/journal.pone.0111715

**Published:** 2014-11-03

**Authors:** Takuya Awata, Hisakuni Yamashita, Susumu Kurihara, Tomoko Morita-Ohkubo, Yumi Miyashita, Shigehiro Katayama, Keisuke Mori, Shin Yoneya, Masakazu Kohda, Yasushi Okazaki, Taro Maruyama, Akira Shimada, Kazuki Yasuda, Nao Nishida, Katsushi Tokunaga, Asako Koike

**Affiliations:** 1 Department of Endocrinology and Diabetes, Faculty of Medicine, Saitama Medical University, Saitama, Japan; 2 Division of RI Laboratory, Biomedical Research Center, Saitama Medical University, Saitama, Japan; 3 Department of Ophthalmology, Faculty of Medicine, Saitama Medical University, Faculty of Medicine, Saitama, Japan; 4 Division of Translational Research, Research Center for Genomic Medicine, Saitama Medical University, Saitama, Japan; 5 Department of Internal Medicine, Saitama Social Insurance Hospital, Saitama, Japan; 6 Department of Internal Medicine, Saiseikai Central Hospital, Tokyo, Japan; 7 Department of Metabolic Disorder, Diabetes Research Center, Research Institute, National Center for Global Health and Medicine, Tokyo, Japan; 8 Research Center for Hepatitis and Immunology, National Center for Global Health and Medicine, Chiba, Japan; 9 Department of Human Genetics, Graduate School of Medicine, The University of Tokyo, Tokyo, Japan; 10 Central Research Laboratory, Hitachi Ltd, Tokyo, Japan; Graduate School of Medicine, University of the Ryukyus, Japan

## Abstract

Elucidation of the genetic susceptibility factors for diabetic retinopathy (DR) is important to gain insight into the pathogenesis of DR, and may help to define genetic risk factors for this condition. In the present study, we conducted a three-stage genome-wide association study (GWAS) to identify DR susceptibility loci in Japanese patients, which comprised a total of 837 type 2 diabetes patients with DR (cases) and 1,149 without DR (controls). From the stage 1 genome-wide scan of 446 subjects (205 cases and 241 controls) on 614,216 SNPs, 249 SNPs were selected for the stage 2 replication in 623 subjects (335 cases and 288 controls). Eight SNPs were further followed up in a stage 3 study of 297 cases and 620 controls. The top signal from the present association analysis was rs9362054 in an intron of *RP1-90L14.1* showing borderline genome-wide significance (*P_met_* = 1.4×10^−7^, meta-analysis of stage 1 and stage 2, allele model). *RP1-90L14.1* is a long intergenic non-coding RNA (lincRNA) adjacent to *KIAA1009/QN1/CEP162* gene; CEP162 plays a critical role in ciliary transition zone formation before ciliogenesis. The present study raises the possibility that the dysregulation of ciliary-associated genes plays a role in susceptibility to DR.

## Introduction

Diabetic retinopathy (DR) remains the most common microvascular complication of diabetes, and is a major cause of visual impairment in adults worldwide [1]. The multifactorial etiology of DR has been postulated, but is poorly understood. Several lines of evidence, such as ethnic differences [2], [3] and familial clustering in both identical twins with type 2 diabetes (T2D) [4] and siblings with either type 1 diabetes [5], [6] or T2D [7]–[9], suggest that genetic factors have a role in the pathogenesis of DR, in addition to the duration of diabetes, hyperglycemia, hypertension and dyslipidemia [10], [11]. Therefore, elucidation of the genetic susceptibility factors for DR is important to gain insight into the pathogenesis of DR, and may define genetic risk factors for this condition.

Initial genetic studies focused on a number of logical candidate genes for DR. Among them, the genes for vascular endothelial growth factor (*VEGFA*), aldose reductase (*AKR1B1*) and the advanced glycation end products receptor (*AGER*) have been the most extensively studied [12]–[14]; in particular, the SNP rs2010963 in the 5′-untranslated region of VEGF, which we first reported had a significant association with DR in 2002 [15], and *AKR1B1* polymorphisms (the dinucleotide repeat microsatellite in the 5′ region and the SNP rs759853 in the promoter region) were confirmed by the meta-analyses performed by Qiu et al. [16] and Abhary et al. [17], respectively.

Subsequently, genome-wide linkage studies on Pima Indians [18], [19] and Mexican-Americans [20] with T2D reported evidence for the linkage of DR to several chromosomal regions, but the susceptibility genes in these regions remain to be elucidated. Recently, several genome-wide association studies (GWAS) in Mexican-Americans [21], Chinese [22], [23] and Caucasians [24] identified multiple susceptibility loci to DR; however, none of these loci reached the genome-wide significance level, and none has been replicated in other studies [14].

In the present study, subsequent to our candidate gene studies for DR in T2D [15], [25]–[27], we conducted a GWAS to identify DR susceptibility loci in approximately two thousand Japanese patients with T2D.

## Materials and Methods

### Ethics Statement

The present study was approved by the Ethical Committee of Saitama Medical University (No. 124-III), the Ethical Committee of Saitama Social Insurance Hospital (No. 00-04), the Ethical Committee of Keio University (No. 13-70-3), the Ethical Committee of the National Center for Global Health and Medicine (No. 75), and the Ethical Committee of University of Tokyo (No. 2541). Written informed consent was obtained from all subjects and all samples were anonymized.

### Genomic DNA Samples and Clinical Data

**Figure 1:**
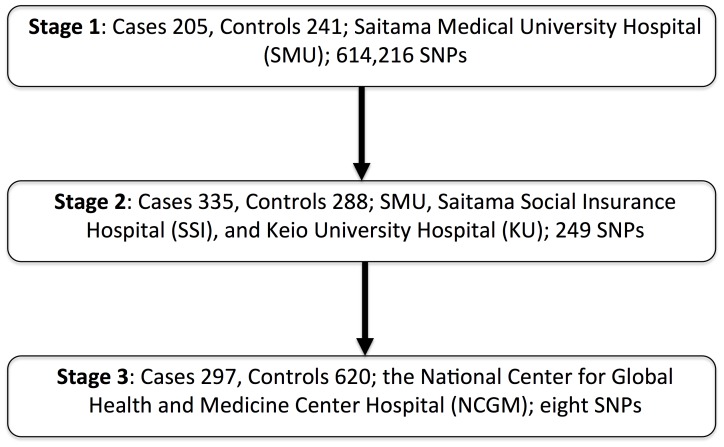
Summary of the study strategy.

The present study applied a three-stage GWAS involving 1,986 Japanese unrelated T2D patients in total (Figure 1). The stage 1 samples for the discovery genome-wide scan were from 446 patients at Saitama Medical University Hospital (SMU); two samples were removed from the initial 448 patients as described below. The stage 2 samples for the replication study of 249 SNPs consisted of those from 623 patients at SMU, the Saitama Social Insurance Hospital (SSI) and Keio University Hospital (KU). The stage 3 samples for the replication study of eight SNPs were from 917 patients at the National Center for Global Health and Medicine Center Hospital (NCGM).

We recruited DNA samples from patients with type 2 diabetes who regularly visited the outpatient clinics (SSI and NCGM) or were admitted to the inpatient wards (SMU and KU) without any particular exclusion criteria. The clinical characteristics of the T2D patients in each group are shown in Table 1. In the present study, the subjects were selected independently from the clinical phenotypes, such as the duration of diabetes and the presence of DR. All patients received fundus examination by trained ophthalmologists, and the severity of DR was classified as none, non-proliferative (NPDR) or proliferative (PDR) based on the international clinical diabetic retinopathy/macular edema disease severity scale. We also collected the data regarding diabetic nephropathy (DN). In the present study, DN was diagnosed by the presence of overt proteinuria or macroalbuminuria (>300 mg/g creatinine).

**Table 1:** Clinical features of Japanese patients with type 2 diabetes.

	Stage 1		Stage 2						Stage 3	
	SMU		SMU		SSI		KU		NCGM	
	DR (+)	DR (−)	DR (+)	DR (−)	DR (+)	DR (−)	DR (+)	DR (−)	DR (+)	DR (−)
n	205	241	118	149	110	78	107	61	297	620
Male Sex (%)	51.2	52.3	49.2^a^	63.1	40.9	46.2	69.2	65.6	62.6	61.0
Age (year)	58.8+−10.8	56.7+−13.7	60.2+−11.7	57.7+−12.2	56.9+−8.5	56.1+−9.5	63.7+−12.0	63.1+−14.2	66.4+−9.5	65.4+−10.7
Duration of diabetes (year)	12.4+−7.7^a^	7.2+−6.5	13.8+−10.6^a^	7.7+−7.2	9.1+−5.6	8.0+−5.8	15.5+−10.5^a^	11.0+−10.5	18.5+−11.5^a^	12.2+−9.9
HbA1c (%)	9.4+−2.0	9.3+−2.2	8.7+−1.9	9.1+−1.9	7.9+−1.3^a^	7.4+−1.3	9.8+−2.0	10.5+−2.1	8.2+−1.7^a^	7.6+−1.6
Hypertension (%)	62.4^a^ (128/205)	52.3 (126/241)	70.3^a^ (83/118)	47.7 (71/149)	65.5^a^ (72/110)	53.2 (41/77)	66.4^a^ (71/107)	42.6 (26/61)	70.3^a^ (196/279)	55.2 (303/549)
Total cholesterol (mg/dL)	195.9+−49.4	194.5+−43.1	188.6+−40.5	195.7+−44.9	199.5+−31.2	202.6+−32.6	204.1+−47.3	207.7+−57.2	189.4+−34.5	192.0+−33.5
Triglycerides (mg/dL)	146.0+−90.4	154.8+−128.1	147.8+−82.6	157.1+−94.4	116.2+−63.9	120.3+−65.9	129.0+−78.9	163.0+−204.9	127.8+−63.0	140.9+−93.6
PDR (%)	39.5 (81/205)	–	57.6 (68/118)	–	12.7 (14/110)	–	30.8 (33/107)	–	19.9 (59/297)	–
DN (%)	24.0^a^ (47/196)	3.5 (8/228)	27.1^a^ (32/118)	4.7 (7/149)	34.5^a^ (38/110)	14.1 (11/78)	36.4^a^ (39/107)	14.8 (9/61)	5.6^a^ (16/284)	0.2 (1/577)

DR: diabetic retinopathy, PDR: proliferative diabetic retinopathy; DN: diabetic nephropathy, SMU: Saitama Medical University Hospital, SSI: Saitama Social Insurance Hospital, KU: Keio University Hospital, NCGM: National Center for Global Health and Medicine Center Hospital.

Data are % or mean +− SD.

a *P*<0.05 vs patients without DR.

### SNP Genotyping and Quality Control

The initial number of samples used in the stage 1 analysis was 448 in total, consisting of 241 patients with DR (cases) and 207 without DR (controls). Genotyping was performed with Affymetrix GeneChip 6.0 microarrays interrogating 909,622 SNPs, according to the manufacturer’s instruction manual. Samples with a genotype call rate of <95%, and SNPs with a genotype call rate of <90%, with a minor allele frequency <0.05 or significant deviation (*P*<0.001) from Hardy-Weinberg equilibrium (HWE) were excluded. Further, population outliers were also excluded based on the principal component analysis (PCA) 1st and 2nd axis values calculated by the EIGENSTRAT software program [28]. Among the stage 1 SNPs showing association of *P*<0.05 with DR under allele model, a total of 249 SNPs were selected for stage 2by the following four criteria (Table S1); (A) 110 SNPs were selected from the regions including the signals of *P*<10^−3^ for DR (allele model), (B) 86 SNP from the regions including the signals of *P*<10^−3^ under other DR-related phenotype (PDR or diabetic macular edema) and/or other model, (C) 29 SNPs with a *P* value between 10^−3^ and 10^−2^ (allele model) and (D) 29 SNPs with a *P* value between 10^−3^ and 10^−2^ (other DR-related phenotype and/or other model) were selected in their biological and/or bioinformatic potency in DR, respectively. For the criteria A and B, one or several SNPs were chosen from those in linkage disequilibrium for each genomic region. We performed cross-platform validation by genotyping 11 SNPs in all stage 1 samples that were run on the Affymetrix GeneChip 6.0 assay using the TaqMan SNP assay, and the concordance rate was 100%.

For the stage 2 replication study, SNP genotyping of a total of 623 patients from SMU, SSI and KU was carried out by the DigiTag2 assay. The DigiTag2 assay was reported to have high accuracy and reproducibility [29]; in addition, a very high concordance rate with the TaqMan SNP assay was also described [30]. Among the 249 selected SNPs, we excluded 24 SNPs with a call rate <95%, and the overall call rate was 99.7%. Eight SNPs were selected for stage 3 based on the overall *P*<10^−3^ combining the stage 1 and stage 2 samples using an allele model. Finally, for the stage 3 replication study, the SNP genotyping of 917 patients from NCGM was carried out using the TaqMan SNP assay. All SNPs had a call rate >98%, and the overall call rate was 99.5%.

### Statistical Analysis

In the comparison of clinical features, continuous clinical data were compared using the unpaired Student’s t-test, and categorical clinical data were compared using Fisher’s exact test. For the stage 1 analysis, the genome-wide association analysis was performed using the PLINK software package (ver. 1.06) [31], R 2.9.0, and the Haploview 1.04 software program [32]. For the stage 2 and 3 replication studies and the combined analyses of the overall samples, the association was tested using the SNPAlyze Ver. 8 Pro software program (Dynacom, Yokohama, Japan). The *P* values were calculated by genotype, allele, dominant, recessive and trend models for each SNP. For the eight selected SNPs from stage 1 and stage 2, logistic regression analysis was also performed to test the association of each SNP with DR, PDR and DN under the genotype (additive), dominant and recessive models adjusted for clinical features. StatView version 5 (SAS Institute, Cary, NC) was used for these tests. A combined meta-analysis was performed using a general variance-based method with a fixed effects model after testing for heterogeneity using Cochran's Q test with R 2.15.2. A total of 614,216 of the 909,622 genome-wide SNPs passed the QC criteria in stage 1. Therefore, the significance level for genome-wide association was considered to be *P* = 8.1×10^−8^ (0.05/614,216). The study power was estimated using the CaTS software program (http://www.sph.umich.edu/csg/abecasis/CaTS/) [33]. The statistical power to detect associations among the present stage 1 (205 cases and 241 controls; 614,216 SNPs) and stage 2 (335 cases and 288 controls; 249 SNPs) was calculated to be 79% or 38%, respectively, based on a genome-wide significance level of *P* = 8.1×10^−8^, the additive genetic model, replication analysis and a genotype relative risk (GRR) of 1.4 or 1.3, assuming a risk allele frequency of 0.40 and disease (DR) prevalence of 50%. In addition, the power to detect stage 1+2 (540 cases and 529 controls; 614,216 SNPs) and stage 3 (297 cases and 620 controls; 8 SNPs) was calculated to be 99% or 85% based on the same conditions and a GRR of 1.4 or 1.3, respectively.

## Results

### Case-Control Association Analysis

A total of 1,986 Japanese patients with type 2 diabetes of the five cohorts, all recruited from Saitama prefecture or the adjacent Tokyo Metropolitan area, were analyzed in the present study. Clinical features of the patients among the cohorts were shown in Table 1; lipid laboratory data were similar, moderate difference was observed in sex, age, duration of diabetes, and substantial difference was observed in HbA1c, presence of PDR and that of DN. Of note, higher HbA1c levels were observed in the SMU (stage 1 and stage 2) and KU (stage 2) patients recruited from the inpatient wards, compared to the SSI (stage 2) and (stage 3) NCGM patients, who were recruited from outpatient clinics.

**Figure 2:**
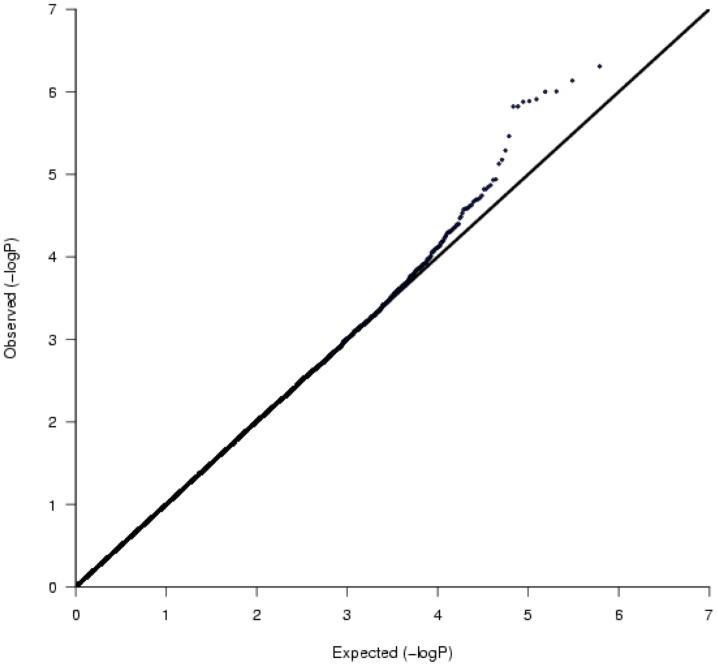
Quantile-quantile plot for the GWAS of diabetic retinopathy in stage 1. *P* values of the Fisher's exact test under the allele model for SNPs in 446 patients were shown. The genomic inflation factor λ was 1.006.

**Figure 3:**
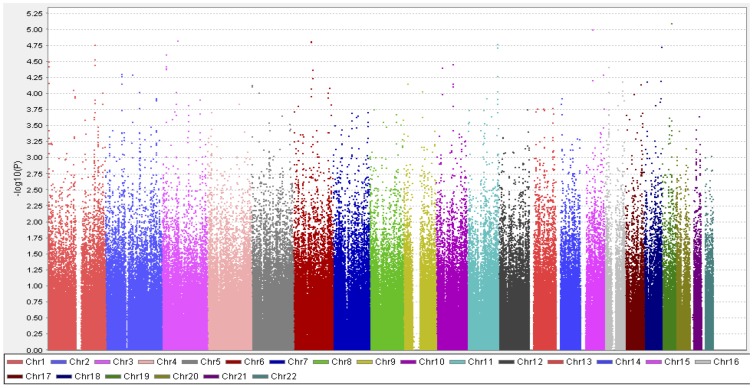
Manhattan plot for the GWAS of diabetic retinopathy in stage 1. *P* values of the Fisher's exact test under the allele model for SNPs in 446 patients were shown.

In stage 1, the association analysis with DR using 614,216 genome-wide SNPs passed the QC criteria, and was originally carried out in 448 Japanese patients recruited from SMU, 207 of whom were DR-positive and 241 of whom were DR-negative. Two DR-positive patients were regarded as outliers by PCA, and were removed from the GWAS (Figure S1). The genomic inflation factor (λ) and mean chi-squared statistics calculated from the GWAS for these 446 patients were 1.006 and 1.003, respectively, both of which were very close to 1.0, suggesting that any observed associations were unlikely to be due to population stratification. The quantile-quantile plot showed apparent deviation from the expected values at the lower *P* values, suggesting that some associations were more significant than expected by chance (Figure 2). The GWAS for DR revealed seven loci (10 SNPs) at a value of *P*<10^−5^ with the best model (Figure 3 and Table S2). The top signals in the present study were at the level of 10^−6^, falling below a genome-wide threshold of *P* = 8.1×10^−8^ (0.05/614,216). The most significant SNP associated with DR was rs4538204, with a *P* value of 1.2×10^−6^ under the recessive model. This SNP is located in an intron of *DPP10* on chromosome 2. The SNPs rs7625611, rs869494, rs4465961 and rs1497516, which were in strong linkage disequilibrium and located in an intergenic region between *AC107622.1* and *ZNF385D* on chromosome 3, were also associated with DR (best *P* = 1.5×10^−6^, dominant model). The remaining five loci associated with DR were located on chromosomes 1, 3, 4, 18 and 19. Among them, the SNPs rs4516615, rs12640858 and rs8107333 are located in introns of *FAM19A1*, *LDB2* and *PCAT19*, respectively.

For the stage 2 replication, 249 SNPs selected from the stage 1 analysis were genotyped in three Japanese cohorts comprising a total of 623 T2D patients, consisting of 267 from SMU (independent cases from the first stage), 188 from SSI and 168 from KU. In an overall analysis combining the stage 1 and stage 2 samples, eight SNPs showed associations with a value of *P*<10^−3^ using the allele model; a meta-analysis combining the stage 1 and stage 2 data using general variance-based method with a fixed effects model showed similar *P* values with no evidence of heterogeneity (Table 2). The most strongly associated SNP, rs9362054 on chromosome 6, reached the genome-wide significance according to the overall analysis (*P* = 4.4×10^−8^), although the level of significance was just above that of genome-wide by the meta-analysis (*P_met_* = 1.4×10^−7^). This SNP is located in the *RP1-90L14.1* gene (see the regional association plot below). The second most significant SNP associated with DR was rs10894267, with a *P_met_* value of 5.8×10^−6^ in an exon of *RP11-890B15.2* on chromosome 11. The remaining six SNPs were weakly associated with DR, with *P_met_* values between 10^−5^ and 10^−3^; two of which were located in introns (*GLIS1* and *CD38*) and four of which were located in intergenic regions.

**Table 2:** SNPs showing associations with diabetic retinopathy (DR) in combined stages 1 and 2 samples with *P*<10^−3^ using the allele model.

													Stage 1+2							Stage 1+2+3					
		RefSeq genes			ENCODE/GENECODE genes				Stage1		Stage2		Overallanalysis		Meta-analysis			Stage3		Overallanalysis			Meta-analysis		
SNP	Chr/Position	Gene	Left gene	Right gene	Gene	Left gene	Right gene	Allele(Risk/Other)	OR	0.399/0.351	OR	*P*	OR	*P*	OR	*P_met_*	*P_het_*	OR	*P*	RiskallelefrequencyDR(+)/DR(−)	OR	*P*	OR	*P_met_*	*P_het_*
rs11582936	1/4909707	NA	*AJAP1*	*MIR4417*	NA	*AJAP1*	*RP11-542C10.1*	C/A	1.71	0.811/0.770	1.25	6.6×10^−2^	1.43	8.4×10^−5^	1.43	8.8×10^−5^	9.0×10^−2^	1.08	6.9×10^−1^	0.399/0.351	1.23	1.9×10^−3^	1.24	1.3×10^−3^	1.7×10^−2^
rs17109215	1/54178200	*GLIS1*	*DMRTB1*	*NDC1*	*GLIS1*	*DMRTB1*	*TMEM48*	A/G	1.56	0.263/0.226	1.41	1.7×10^−2^	1.46	4.3×10^−4^	1.47	3.9×10^−4^	6.5×10^−1^	1.08	5.6×10^−1^	0.811/0.770	1.28	2.4×10^−3^	1.28	2.2×10^−3^	1.5×10^−1^
rs4580644	4/15785201	*CD38*	*BST1*	*FGFBP1*	*CD38*	*BST1*	*FGFBP1*	T/C	1.62	0.347/0.305	1.41	1.1×10^−2^	1.51	5.7×10^−5^	1.50	7.4×10^−5^	4.9×10^−^1	0.88	3.0×10^−1^	0.263/0.226	1.22	6.9×10^−3^	1.20	1.8×10^−2^	2.9×10^−3^
rs12641981	4/45179883	NA	*GNPDA2*	*GABRG1*	NA	*RP11-362I1.1*	*U6*	T/C	1.52	0.602/0.552	1.58	9.3×10^−3^	1.42	2.2×10^−4^	1.45	8.1×10^−5^	6.5×10^−1^	1.00	9.7×10^−1^	0.347/0.305	1.21	5.3×10^−3^	1.23	3.3×10^−3^	2.8×10^−2^
rs1156082	6/77501140	NA	*IMPG1*	*HTR1B*	NA	*RP11-35J1.1*	*HTR1B*	C/T	1.69	0.365/0.291	1.29	3.2×10^−2^	1.45	3.4×10^−5^	1.44	3.8×10^−5^	1.3×10^−^1	0.97	7.8×10^−1^	0.602/0.552	1.21	4.0×10^−3^	1.21	3.7×10^−3^	4.3×10^−3^
rs9362054	6/85178268	NA	*CEP162*	*TBX18*	*RP1-90L14.1*	*KIAA1009*	*RP11-132M7.1*	T/C	1.84	0.867/0.846	1.52	7.1×10^−4^	1.67	4.4×10^−8^	1.64	1.4×10^−7^	3.2×10^−1^	1.06	6.2×10^−1^	0.365/0.291	1.40	1.1×10^−6^	1.36	1.7×10^−5^	5.6×10^−3^
rs7083364	10/27196099	NA	*ABI1*	*LINC00202-1*	NA	*snoU13*	*LINC00202-1*	T/G	2.36	0.564/0.518	1.52	2.8×10^−2^	1.83	1.0×10^−5^	1.83	1.4×10^−5^	1.2×10^−1^	0.77	5.6×10^−2^	0.867/0.846	1.19	7.3×10^−2^	1.18	8.8×10^−2^	1.7×10^−5^
rs10894267	11/130714861	NA	*C11orf44*	*SNX19*	*RP11-890B15.2*	*C11orf44*	*RP11-890B15.3*	T/C	1.78	2.8×10^−5^	1.32	1.8×10^−2^	1.49	5.5×10^−6^	1.49	5.8×10^−6^	9.3×10^−2^	0.95	6.0×10^−1^	0.564/0.518	1.20	4.4×10^−3^	1.22	2.2×10^−3^	8.0×10^−4^

These eight SNPs were selected for the stage 3 replication of genotyping in 917 T2D patients from the NCGM. As shown in Table 3, a meta-analysis combining all three stages revealed that the association of SNP rs9362054 with DR was the strongest, with a *P_met_* value of 1.7×10^−5^ using the allele model, although significant heterogeneity was observed for *P_het_* = 5.6×10^−3^. The other seven SNPs all showed weak associations, with *P* values higher than 10^−3^ (Table 2).

**Table 3:** Associations of rs9362054 in *RP1-90L14.1* with diabetic retinopathy (DR), proliferative diabetic retinopathy (PDR) and diabetic nephropathy (DN) in each stage and combined stages 1, 2 and 3, using logistic regression under genotype, dominant and recessive models after adjusting for sex, duration of diabetes and HbA1c.

	DR																PDR					DN				
					Stage 1+2							Stage 1+2+3					Stage 1+2+3					Stage 1+2+3				
	Stage 1		Stage 2		Overallanalysis		Meta-analysis			Stage 3		Overallanalysis		Meta-analysis			Overallanalysis		Meta-analysis			Overallanalysis		Meta-analysis		
	OR	*P*	OR	*P*	OR	*P*	OR	*P_met_*	*P_het_*	OR	*P*	OR	*P*	OR	*P_met_*	*P_het_*	OR	*P*	OR	*P_met_*	*P_het_*	OR	*P*	OR	*P_met_*	*P_het_*
genotype model^a^	1.59	2.1×10^−3^	1.59	2.4×10^−4^	1.63	3.4×10^−7^	1.59	1.8×10^−6^	1.0×10^0^	1.15	2.5×10^−1^	1.45	1.7×10^−7^	1.39	4.8×10^−6^	1.3×10^−1^	1.21	6.1×10^−2^	1.12	2.6×10^−1^	6.4×10^−2^	1.68	1.0×10^−6^	1.53	1.2×10^−4^	4.8×10^−1^
dominant model	1.54	3.5×10^−2^	1.67	3.7×10^−3^	1.68	9.3×10^−5^	1.61	1.9×10^−4^	7.5×10^−1^	1.11	5.0×10^−1^	1.41	4.1×10^−4^	1.36	1.2×10^−3^	1.3×10^−1^	1.23	1.4×10^−1^	1.17	2.7×10^−1^	3.5×10^−1^	1.85	9.3×10^−5^	1.79	4.4×10^−4^	4.8×10^−1^
recessive model	3.00	1.2×10^−3^	2.27	1.2×10^−3^	2.60	2.3×10^−6^	2.51	6.0×10^−6^	5.1×10^−1^	1.46	1.5×10^−1^	2.32	3.3×10^−8^	2.14	1.3×10^−6^	3.8×10^−1^	1.39	1.0×10^−1^	1.20	3.9×10^−1^	3.0×10^−2^	2.27	2.4×10^−5^	1.78	5.6×10^−3^	6.5×10^−1^

a With additive genetic effect.

The association of SNP rs9362054 was further evaluated by multiple logistic regression analysis using genotype, dominant and recessive models. After controlling for sex, the duration of diabetes and the HbA1c level, SNP rs9362054 was found to be strongly associated with DR according to a an overall analysis or meta-analysis (Table 3); controlling for hypertension and the lipid laboratory data was also performed with similar results (data not shown). The most significant signal was obtained in an overall analysis combining all stages in the recessive model (3.3×10^−8^) with the genome-wide significance level, while the meta-analysis of all stages showed a *P_met_* value of 1.3×10^−6^ with no evidence of heterogeneity (*P_het_* = 3.8×10^−1^). Regarding the associations with PDR and DN as determined by multiple logistic regression analysis of all stages (Table 3), rs9362054 was not significantly associated with PDR under all models, but was significantly associated with DN with *P_met_* = 1.2×10^−4^ under the genotype model. Furthermore, according to a logistic regression analysis including the severity of DR (i.e. DR-negative, NPDR or PDR) as a variable, rs9362054 was significantly associated with DN (*P_met_* = 3.5×10^−3^ under the genotype model) independent of the severity of DR.

### Regional Association Plot Around rs9362054

**Figure 4:**
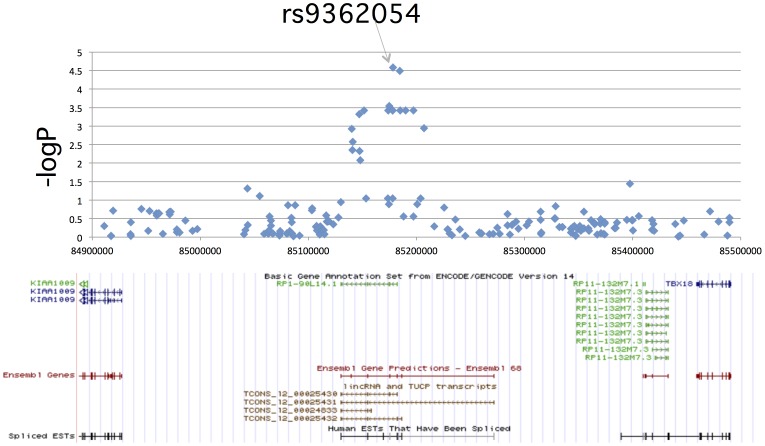
Regional association plot of the region around the SNP rs9362054. The -log_10_P values of the Fisher's exact test under the allele model in the GWAS of diabetic retinopathy in stage 1 were plotted against relative chromosomal locations (UCSC human genome 19).

The most DR-associated SNP rs9362054 was located at 85Mb on chromosome 6. This SNP is in an intron of the *RP1-90L14.1* gene between two protein-coding genes, *KIAA1009* and *TBX18*. According to the UCSC human genome 19, *RP1-90L14.1* is annotated as a long intergenic non-coding RNA (lincRNA) [34] by the GENECODE project. The regional plots for SNP rs9362054 demonstrated that the association signals with *P*<0.01 were confined to the *RP1-90L14.1* gene region (Figure 4).

## Discussion

In the present study, we performed a three-stage GWAS for DR in Japanese T2D patients. The top SNP, rs9362054, was located in an intron of the *RP1-90L14.1* gene; regional plots showed that the association signals around rs9362054 were confined to this gene region. *RP1-90L14.1* encodes a lincRNA, a class of long non-coding RNAs (lncRNAs) [34], [35] that are transcribed from intergenic regions; three transcripts were mapped to this gene as splice variants, and six non-coding exons were estimated to be present by the Ensembl project. The *RP1-90L14.1* locus is widely conserved among vertebrates (Human, Rhesus, Mouse, Dog, Elephant, Opossum, Chicken, *Xenopus tropicalis* and Zebrafish) according to the UCSC Genome Browser. RP1-90L14.1 is also called lnc-KIAA1009-1 by LNCipedia 2.0 (a long non-coding RNA database) [36], because the nearest protein-coding gene on the same strand is *KIAA1009*; it is possible that *RP1-90L14.1/lnc-KIAA1009-1* and *KIAA1009* are functionally connected, since cis-regulation has been shown for many lncRNA genes [35].

Interestingly, KIAA1009, also known as QN1, was originally reported to be involved in cell cycle control during retinal development [37], and was found to be a microtubule-associated ATPase involved in cell division [38]. Recently, it has been revealed that KIAA1009, renamed CEP162, plays a critical role in ciliary transition zone formation before ciliogenesis [39]. Ciliopathies [40], [41] comprise a group of disorders associated with dysfunction of a hair-like cellular organelle called a cilium; as cilia are a component of almost all vertebrate cells, cilial dysfunction can manifest as a constellation of features that include, characteristically, retinal degeneration, renal disease and cerebral anomalies; retinal degeneration is a common feature of ciliopathic disorders [42], [43], and may be associated with retinal cell death [40]. In this regard, the strong signal for the *RP1-90L14.1/lnc-KIAA1009-1* locus in the present study could be mediated through defective ciliogenesis due to KIAA1009/QN1/CEP162 dysregulation by some *lnc-KIAA1009-1* variation(s). Although we should be cautious regarding this hypothesis, it was previously reported that transgenic rats with overexpression of a mutant cilia gene, polycystin-2, exhibited similar specific phenotypes to diabetic retinopathy, such as vasoregression and pericyte loss [44]: furthermore, in the same transgenic rats, streptozotocin-induced chronic hyperglycemia was shown to ameliorate vasoregression and neuroglia cell loss [45], in agreement with the finding of a weaker association between *RP1-90L14.1* and PDR than with DR (Table 3). In addition, it was noted that the previous GWAS for DR in Chinese patients [23] identified a signal in the locus of *LRP-BBS5*, and BBS5 is also known to be important in ciliogenesis [40].

In agreement with the frequent involvement of both retinopathy and renal disease in ciliopathies [40], [41], [46], rs9362054 in *RP1-90L14.1* was also significantly associated with DN (Table 3). It should be noted that a significant association with DN was observed independently from the severity of DR, suggesting that rs9362054 may also contribute to the development of DN. To our knowledge, there has been no previous study reporting the association between DN and ciliary genes. Since the present study was not designed to identify DN susceptibility genes, further well-designed studies are required to verify the existence of any possible associations between the ciliary genes and DN.

In the present study, the stage 3 replications were mostly negative. However, possibly due to clinical heterogeneity (such as the duration of diabetes, metabolic control and medication(s) used for diabetes), the modest sample size and some bias, the replication of associations with DR appears to be difficult, as evidenced by the previous GWAS studies [14]; i.e. these studies have reported only borderline associations, and there were no obvious overlapping susceptibility loci between studies. Furthermore, in the present study, the potential susceptibility loci (Table S1 and Table 2) did not overlap.

One of the strengths of the present study is considered to be that the homogeneity of the subjects was expected because the participants were recruited exclusively from the Kanto district (Saitama prefecture or the adjacent Tokyo Metropolitan area), Japan. Furthermore, we took into account various clinical features, including the duration of diabetes and the HbA1c level, and the associations with PDR or DN were also evaluated in comparison to that with DR.

There are several limitations regarding the present study that should be kept in mind. First, the modest sample size of the stage 1 discovery cohort had a limited power to detect susceptibility SNPs; in fact, none reached the genome-wide significance level (*P* = 8.1×10^−8^). Consequently, the SNPs selected based on both provisional significance (*P*<10^−3^) and the viewpoint of the biological and/or bioinformatics potency in DR were proceeded for the stage 2 replication, and the two-stage analysis of stage 1 and stage 2 had sufficient power to detect susceptibility SNPs with relatively a high effect. Second, the documented HbA1c levels were collected at only one point, and were considerably different among the cohorts. Third, the patients with a short disease duration were not excluded from the control group, which may have reduced the power of the study, although the real duration of type 2 diabetes is considered to be longer than the clinically defined duration by several years. Finally, the stage 3 replication was carried out, although no associations of the stage 1+2 SNPs were confirmed despite the adequate power, as described in the statistical analysis section. In addition, under the predefined significance threshold of 1.6×10^−4^ (2×8.1×10^−8^/10^−3^) according to the replication-based method, the stage 3 analysis of rs9362054 had 85% power to detect association with the genome-wide significance using GDesignPlus [47] (StaGen Co., Ltd., Tokyo, Japan). Rather than the possibility of a false positive finding, we speculate that the substantial heterogeneity between the stage 3 and stage 1+2 studies (Table 2), as evidenced by the fact that the HbA1c levels were relatively low and the proportion of DR was the lowest among the cohorts in the stage 3 patients could have weakened the contribution of genetic factors to the development of DR. Recently, it has been demonstrated that a substantial proportion of the associations with borderline genome-wide significance are replicable [48]. Therefore, further case-control studies of large well-characterized cohorts would be useful for validating the findings of the present study.

In conclusion, in the present GWAS in Japanese T2D patients, the most DR-associated locus, the lincRNA *RP1-90L14.1*, is intriguing, since the adjacent *CEP162* gene plays an important role in ciliogenesis. Taking into account the findings in previous studies of the transgenic rats and the GWAS in Chinese, the present study raises the possibility that the dysregulation of ciliary-associated genes plays a role in susceptibility to DR.

## Supporting Information

Figure S1 **Principal component analysis for the GWAS of diabetic retinopathy in stage 1.** The results were shown along with Japanese (JPT) and Han-Chinese (CHB) individuals in the HapMap database. Two subjects were regarded as outliers, and were removed from the present study.
(TIFF)

Table S1 **SNPs selected from stage 1 genome-wide scan.** DR: diabetic retinopathy, PDR: proliferative diabetic retinopathy, DME: diabetic macular edema.
(XLSX)

Table S2 **Stage 1 SNPs showing associations with diabetic retinopathy with** ***P*** **<10^−5^ using the best model.**
(DOCX)
