# Correction: A Genome-Wide Association Study for Diabetic Retinopathy in a Japanese Population: Potential Association with a Long Intergenic Non-Coding RNA

**DOI:** 10.1371/journal.pone.0126789

**Published:** 2015-04-24

**Authors:** Takuya Awata, Hisakuni Yamashita, Susumu Kurihara, Tomoko Morita-Ohkubo, Yumi Miyashita, Shigehiro Katayama, Keisuke Mori, Shin Yoneya, Masakazu Kohda, Yasushi Okazaki, Taro Maruyama, Akira Shimada, Kazuki Yasuda, Nao Nishida, Katsushi Tokunaga, Asako Koike

There are errors in Tables 2 and S2. Please see the corrected tables below.

**Table 2:** SNPs showing associations with diabetic retinopathy (DR) in combined stages 1 and 2 samples with *P*<10^–3^ using the allele model.

													Stage 1+2							Stage 1 + 2 + 3					
		RefSeq genes			ENCODE/GENECODE genes				Stage 1		Stage 2		Overall analysis		Meta-analysis			Stage 3		Overall analysis			Meta-analysis		
SNP	Chr / Position	Gene	Left gene	Right gene	Gene	Left gene	Right gene	Allele (Risk / Other)	OR	*P*	OR	*P*	OR	*P*	OR	*P*_*met*_	*P*_*het*_	OR	*P*	Risk allele frequencyDR(+)/DR(-)	OR	*P*	OR	*P*_*met*_	*P*_*het*_
rs11582936	1/4909707	NA	*AJAP1*	*MIR4417*	NA	*AJAP1*	*RP11-542C10*.*1*	C/A	1.71	1.5 x 10^–4^	1.25	6.6 x 10^–2^	1.43	8.4 x 10^–5^	1.43	8.8 x 10^–5^	9.0 x 10^–2^	1.08	6.9 x 10^–1^	0.399/0.351	1.23	1.9 x 10^–3^	1.24	1.3 x 10^–3^	1.7 x 10^–2^
rs17109215	1/54178200	*GLIS1*	*DMRTB1*	*NDC1*	*GLIS1*	*DMRTB1*	*TMEM48*	A/G	1.56	1.0 x 10^–2^	1.41	1.7 x 10^–2^	1.46	4.3 x 10^–4^	1.47	3.9 x 10^–4^	6.5 x 10^–1^	1.08	5.6 x 10^–1^	0.811/0.770	1.28	2.4 x 10^–3^	1.28	2.2 x 10^–3^	1.5 x 10^–1^
rs4580644	4/15785201	*CD38*	*BST1*	*FGFBP1*	*CD38*	*BST1*	*FGFBP1*	T/C	1.62	2.2 x 10^–3^	1.41	1.1 x 10^–2^	1.51	5.7 x 10^–5^	1.50	7.4 x 10^–5^	4.9 x 10^–^1	0.88	3.0 x 10^–1^	0.263/0.226	1.22	6.9 x 10^–3^	1.20	1.8 x 10^–2^	2.9 x 10^–3^
rs12641981	4/45179883	NA	*GNPDA2*	*GABRG1*	NA	*RP11-362I1*.*1*	*U6*	T/C	1.52	3.2 x 10^–3^	1.58	9.3 x 10^–3^	1.42	2.2 x 10^–4^	1.45	8.1 x 10^–5^	6.5 x 10^–1^	1.00	9.7 x 10^–1^	0.347/0.305	1.21	5.3 x 10^–3^	1.23	3.3 x 10^–3^	2.8 x 10^–2^
rs1156082	6/77501140	NA	*IMPG1*	*HTR1B*	NA	*RP11-35J1*.*1*	*HTR1B*	C/T	1.69	1.4 x 10^–4^	1.29	3.2 x 10^–2^	1.45	3.4 x 10^–5^	1.44	3.8 x 10^–5^	1.3 x 10^–^1	0.97	7.8 x 10^–1^	0.602/0.552	1.21	4.0 x 10^–3^	1.21	3.7 x 10^–3^	4.3 x 10^–3^
rs9362054	6/85178268	NA	*CEP162*	*TBX18*	*RP1-90L14*.*1*	*KIAA1009*	*RP11-132M7*.*1*	T/C	1.84	4.1 x 10^–5^	1.52	7.1 x 10^–4^	1.67	4.4 x 10^–8^	1.64	1.4 x 10^–7^	3.2 x 10^–1^	1.06	6.2 x 10^–1^	0.365/0.291	1.40	1.1 x 10^–6^	1.36	1.7 x 10^–5^	5.6 x 10^–3^
rs7083364	10/27196099	NA	*ABI1*	*LINC00202-1*	NA	*snoU13*	*LINC00202-1*	T/G	2.36	3.5 x 10^–5^	1.52	2.8 x 10^–2^	1.83	1.0 x 10^–5^	1.83	1.4 x 10^–5^	1.2 x 10^–1^	0.77	5.6 x 10^–2^	0.867/0.846	1.19	7.3 x 10^–2^	1.18	8.8 x 10^–2^	1.7 x 10^–5^
rs10894267	11/130714861	NA	*C11orf44*	*SNX19*	*RP11-890B15*.*2*	*C11orf44*	*RP11-890B15*.*3*	T/C	1.78	2.8 x 10^–5^	1.32	1.8 x 10^–2^	1.49	5.5 x 10^–6^	1.49	5.8 x 10^–6^	9.3 x 10^–2^	0.95	6.0 x 10^–1^	0.564/0.518	1.20	4.4 x 10^–3^	1.22	2.2 x 10^–3^	8.0 x 10^–4^

## Supporting Information

S2 Table Stage 1 SNPs showing associations with diabetic retinopathy with *P*<10^–5^ using the best model. (DOCX)
