# Supplementary figures and images for: A Genome-Wide Association Study for Diabetic Retinopathy in a Japanese Population: Potential Association with a Long Intergenic Non-Coding RNA
Source: PLoS One. 2014 Nov 3;9(11):e111715. doi: 10.1371/journal.pone.0111715 (PMC4218806; doi:10.1371/journal.pone.0111715)

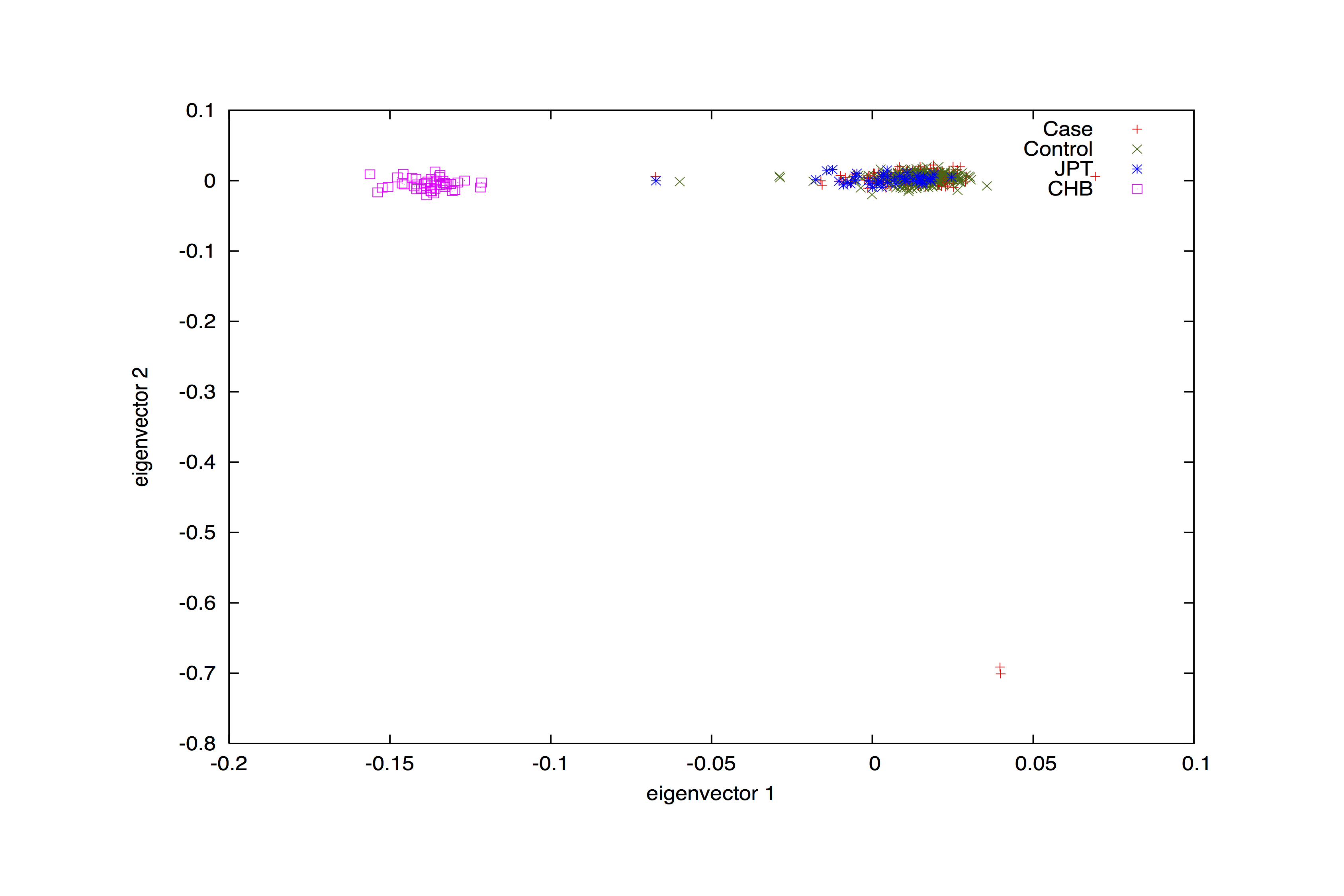

Supplement: Figure S1 — Principal component analysis for the GWAS of diabetic retinopathy in stage 1. The results were shown along with Japanese (JPT) and Han-Chinese (CHB) individuals in the HapMap database. Two subjects were regarded as outliers, and were removed from the present study. (TIFF) [file pone.0111715.s001.tiff]
