# Supplementary material for: Correction: A Genome-Wide Association Study for Diabetic Retinopathy in a Japanese Population: Potential Association with a Long Intergenic Non-Coding RNA
Source: PLoS One. 2015 Apr 24;10(4):e0126789. doi: 10.1371/journal.pone.0126789 (PMC4409015; doi:10.1371/journal.pone.0126789)
Supplement: S2 Table — (DOCX) [file pone.0126789.s001.docx]

**Table S2.** Stage 1 SNPs showing associations with diabetic retinopathy with *P*<10^-5^ using the best model.

|  |  | RefSeq genes | | | ENCODE/GENECODE genes | | | *P* value | | | | |
| --- | --- | --- | --- | --- | --- | --- | --- | --- | --- | --- | --- | --- |
| SNP | Chr/Position | Gene | Left gene | Right gene | Gene | Left gene | Right gene | Genotype | Allele | Dominant | Recessive | Trend |
| rs10920631 | 1/203367047 | NA | *FMOD* | *PRELP* | NA | *FMOD* | *PRELP* | 5.3 x 10^-5^ | 1.1 x 10^-5^ | 1.0 x 10^-5^ | 6.4 x 10^-2^ | 2.9 x 10^-5^ |
| rs4538204 | 2/116393666 | *DPP10* | *LINC01191* | *DDX18* | *DPP10* | *U2* | *RNU7-190P* | 4.8 x 10^-6^ | 4.8 x 10^-5^ | 6.9 x 10^-2^ | 1.2 x 10^-6^ | 7.4 x 10^-5^ |
| rs7625611 | 3/21027910 | NA | *AC107622.1* | *VENTXP7^a^/ ZNF385D* | NA | *U6* | *AC104441.1* | 1.2 x 10^-5^ | 2.9 x 10^-5^ | 3.1 x 10^-6^ | 5.3 x 10^-1^ | 3.1 x 10^-5^ |
| rs869494 | 3/21061473 | NA | *AC107622.1* | *VENTXP7^a^/ ZNF385D* | NA | *U6* | *AC104441.1* | 1.1 x 10^-5^ | 3.5 x 10^-5^ | 3.8 x 10^-6^ | 6.1 x 10^-1^ | 2.2 x 10^-5^ |
| rs4465961 | 3/21063058 | NA | *AC107622.1* | *VENTXP7^a^/ ZNF385D* | NA | *U6* | *AC104441.1* | 4.2 x 10^-6^ | 1.7 x 10^-5^ | 1.5 x 10^-6^ | 6.1 x 10^-1^ | 1.2 x 10^-5^ |
| rs1497516 | 3/21065354 | NA | *AC107622.1* | *VENTXP7^a^/ ZNF385D* | NA | *U6* | *AC104441.1* | 1.6 x 10^-5^ | 2.7 x 10^-5^ | 4.2 x 10^-6^ | 4.5 x 10^-1^ | 2.0 x 10^-5^ |
| rs4516615 | 3/68344674 | *FAM19A1* | *SUCLG2-AS1* | *FAM19A4* | *FAM19A1* | *RP11-81N13.1* | *AC096922.1* | 1.1 x 10^-5^ | 2.4 x 10^-5^ | 8.3 x 10^-6^ | 1.0 | 2.9 x 10^-5^ |
| rs12640858 | 4/16541432 | *LDB2* | *TAPT1-AS1* | *QDPR* | *LDB2* | *RP11-446J8.1* | *RP11-141E13.1* | 1.4 x 10^-5^ | 1.5 x 10^-4^ | 3.9 x 10^-2^ | 3.5 x 10^-6^ | 1.3 x 10^-4^ |
| rs11878142 | 18/76004155 | NA | *LINC01029* | *SALL3* | NA | *RP11-100K18.1* | *RP11-451L19.1* | 1.7 x 10^-5^ | 1.7 x 10^-5^ | 5.6 x 10^-6^ | 2.3 x 10^-2^ | 6.9 x 10^-6^ |
| rs8107333 | 19/41987873 | *PCAT19* | *ER1CH4* | *AC006129.4* | *AC011526.1* | *C19orf69* | *AC006129.1* | 3.6 x 10^-5^ | 7.8 x 10^-6^ | 2.6 x 10^-4^ | 3.4 x 10^-4^ | 6.8 x 10^-6^ |

^a^pseudogene.
